# Transmagnetic Stimulation in Special Situations: Is Its Use Safe During Pregnancy?

**DOI:** 10.1192/j.eurpsy.2025.205

**Published:** 2025-08-26

**Authors:** P. Lusilla

**Affiliations:** Psiquiatria, Hospital Universitario Vall d’Hebron, Barcelona, Spain

## Abstract

**Abstract:**

Transcranial Magnetic Stimulation (TMS) is a non-invasive procedure that uses magnetic fields to stimulate nerve cells in the brain. It is primarily used to treat depression and other mental health conditions. When it comes to the safety of TMS during pregnancy, the current evidence is limited but generally suggests that it may be a safer alternative to medications that could potentially harm the fetus. Several small studies and case reports have indicated that TMS does not appear to pose significant risks to the pregnant woman or the fetus. However, the data is not extensive, and more research is needed to fully understand the implications. The procedure is typically avoided in the first trimester unless absolutely necessary, as this is a critical period for fetal development. In summary, while TMS is considered relatively safe during pregnancy, especially compared to some pharmacological treatments, it should only be used when the potential benefits outweigh the risks, and under the close supervision of a healthcare provider. Pregnant women considering TMS should discuss their specific situation with their doctor to make an informed decision.

**Disclosure of Interest:**

None Declared

